# Melatonin Acts as Antioxidant and Improves Sleep in MS Patients

**DOI:** 10.1007/s11064-014-1347-6

**Published:** 2014-06-30

**Authors:** Monika Adamczyk-Sowa, Krystyna Pierzchala, Pawel Sowa, Sebastian Mucha, Izabela Sadowska-Bartosz, Jowita Adamczyk, Marcin Hartel

**Affiliations:** 1 Department of Neurology in Zabrze, Medical University of Silesia, ul. 3-go Maja 13-15, 41-800 Zabrze, Poland; 2 ENT Department in Zabrze, Medical University of Silesia, Zabrze, Poland; 3 Department of Biochemistry and Cell Biology, University of Rzeszow, ul. Zelwerowicza 4, 35-601 Rzeszow, Poland; 4 Department of Physiology in Zabrze, Medical University of Silesia, Zabrze, Poland; 5 MCD Voxel, MRI Department, Zabrze, Poland

**Keywords:** Melatonin, Multiple sclerosis, TAC, TOS, Sleep, Athens Insomnia Scale

## Abstract

The relationship between the prevalence of multiple sclerosis (MS) and sunlight’s ultraviolet radiation was proved. Oxidative stress plays a role in the pathogenic traits of MS. Melatonin possesses antioxidative properties and regulates circadian rhythms. Sleep disturbances in MS patients are common and contribute to daytime fatigue. The aim of study was to evaluate 5 mg daily melatonin supplementation over 90 days on serum total oxidant status (TOS), total antioxidant capacity (TAC) and its influence on sleep quality and depression level of MS patients. A case–control prospective study was performed on 102 MS patients and 20 controls matched for age and sex. The Kurtzke’s Expanded Disability Status Scale, magnetic resonance imaging examinations, Athens Insomnia Scale (AIS), Beck Depression Inventory questionnaires were completed. Serum TOS and TAC levels were measured. We observed higher serum levels of TOS in all MS groups, while after melatonin treatment the TOS levels significantly decreased. The TAC level was significantly lower only in mitoxantrone-treated group and it increased after melatonin supplementation. A strong positive correlation between T1Gd(+) number lesions and TAC level in interferon-beta-1A group was observed. AIS group mean score above 6 defining insomnia were observed in interferon-beta-1B-group, glatiramer acetate-group and mitoxantrone-group: 6.62 ± 2.88, 8.45 ± 2.07, 11.1 ± 3.25, respectively. After melatonin treatment the AIS mean scores decrease in glatiramer acetate-group and mitoxantrone-group achieving 5.25 ± 1.14 and 7.08 ± 2.39, respectively (*p* < 0.05). Finding from our study suggest that melatonin can act as an antioxidant and improves reduced sleep quality in MS patients.

## Introduction

Multiple sclerosis (MS) is an immune-mediated, the most prevalent demyelinating disease of the central nervous system (CNS) in young adults. Environmental factors play an important role in MS etiology. The relationship between the prevalence of MS, latitude gradient, and sunlight’s ultraviolet radiation was proved [1]. MS incidence is higher in geographic regions with sunlight exposure deficiency. Melatonin and vitamin D are mediators of the effects of sunlight in healthy individuals, and both are thought to play a role in MS pathophysiology [1, 2].

To date, the exact mechanisms of MS pathophysiology remain to be fully explained, but a triad of neural tissue injury mechanisms: inflammation, demyelination, and axonal damage is still valid [3]. Recent studies have suggested that oxidative stress can play a crucial role in the pathogenic traits of MS. The CNS is very susceptible to oxidative damage [4, 5].

Melatonin (*N*-acetyl-5-methoxytryptamine) is a natural hormone mainly produced in the mammalian pineal gland during the dark phase. With its lipophilic and hydrophilic character, melatonin freely crosses the blood–brain barrier and enters all cells. Melatonin acts through G-protein coupled membrane receptors, MT1 and MT2 and through nuclear receptors RZR/ROR [6]. Both receptors are widely present in the central and peripheral nervous systems, and have been associated with cell differentiation and immune response regulation. Moreover, melatonin receptors are expressed on the membrane of CD4 T cells, CD8 T cells, and B cells [7]. Melatonin and its metabolites have been demonstrated to possess multiple functions, including antioxidation, immunomodulatory and anti-inflammatory effects [3, 8].

Melatonin influences antioxidative defensive systems, up-regulates gene expression, and stimulates the activities of several antioxidant enzymes, including glutathione peroxidase (GSH-Px), superoxide dismutase (SOD), catalase (CAT), as well as the levels of glutathione (GSH) [8–11]. Melatonin has been shown to have immunomodulatory properties involved in the regulation of both the cellular and humoral immunity. The regulatory function of melatonin on immune mechanisms is seasonally dependent. As a consequence, melatonin improves the clinical course of illnesses with inflammatory etiology [3, 12]. Moreover, it participates in neurogenesis, regulation of circadian rhythms, sleep, and also exerts an anti-tumor activity [3, 12, 13].

The important role of melatonin as a modulator of sleep is well documented. Melatonin treatment can also entrain the circardian clock and could be helpful in coping with jet-lag and shift-work, as well as be useful in blind subjects, and in individuals with delayed or advanced sleep phase disorders. Administration of exogenous melatonin has been reported to increase sleep quality and length, and decrease sleep latency and the number of wakeful episodes in elderly women with insomnia, as well as significantly improve functioning the following day [12]. It is known that sleep disturbances are present more often in MS patients than in the general population and contribute to daytime fatigue [14, 15]. There are only few original papers concerning sleep problems in MS patients. The prevalence of sleep problems was reported in 24–50 % of MS patients [16, 17]. The possible common pathomechanisms shared by MS and sleep disturbances may be connected with circadian rhythm disorders with compromised melatonin secretion reducing input to the suprachiasmatic nucleus due to impaired visual pathways, as well as increased levels of proinflammatory cytokines [18].

The role of melatonin in MS has been suggested in several clinical studies. It was found that shift work at a young age increases the risk of MS [19]. Melatonin levels in saliva were significantly low among patients with MS after controlling the effect of age. [20]. Moreover, it was proved that melatonin and its immediate precursor *N*-acetylserotonin decrease demyelination and increase remyelination under inflammatory conditions [21]. It was reported that MS is associated with increased levels of depression, which may lead to decreases in melatonin levels. It is known that depression is also linked to sleep dysregulation. Moreover depression is associated with decreased levels of serotonin, which is a precursor for melatonin [22].

The aim of our present study was to evaluate the action of melatonin as a molecule with antioxidative properties on the total oxidant status (TOS) in serum and total antioxidant capacity (TAC) levels in patients with MS, a disease with well-documented oxidative stress disturbance pathology. Moreover, we also intended to ascertain the influence of melatonin on sleep quality and depression levels of MS patients.

## Materials and Methods

### Patients

A case–control prospective study was performed on 102 MS diagnosed according to the McDonald criteria (2005) [23] patients (69 women, 33 men) and 20 (13 women, 7 men) healthy subjects matched for age and sex, observed in 2013 in the Department of Neurology in Zabrze, Medical University of Silesia, Poland. The patients were divided into following groups:

- *Group C* (*control group*) 20 healthy controls observed in our Department due to undiagnosed headaches. Controls were matched for age and sex with the study group.
- *Group P* (*pre*-*treated MS group*) 21 patients with *de novo* relapsing-remitting form of MS (RRMS), without any immunomodifying MS treatment.
- *Group A* (*beta*-*1a interferons treated MS group*) 25 patients with RRMS. All of them received interferon beta-1a, applied once per week as an intramuscular injection.
- *Group B* (*beta*-*1b interferons treated MS group*) 27 patients with RRMS. All of them received interferon beta-1b, injected subcutaneously every other day.
- *Group G* (*glatiramer acetate treated MS group*) 12 patients with RRMS. All of them received daily subcutaneous glatiramer acetate injections.
- *Group MX* (*mitoxantrone treated MS group*) 17 patients with secondarily progressive (SP) or progressive-relapsing (PR) form of MS. All of them received 5 doses of mitoxantrone *iv* (12 mg/m^2^/dose) administered quarterly.

None of the patients received any antioxidative substances, vitamins, anti-inflammatory, or hormonal treatment for at least 3 months prior to the study, and any sleeping pills 2 weeks prior to the study.

Demographic characteristics of the patients were presented in Table 1.

**Table 1:** Demographic characteristics of studied subjects divided into groups: A (beta-1a interferons treated RRMS group), B (beta-1b interferons treated RRMS group), G (glatiramer acetate treated RRMS group), MX (mitoxantrone treated SP or PR MS group), P (immunomodifying pre-treated RRMS group), C (control healthy group)

Group	A	B	G	MX	P	Control
Patients number (n) (total n = 122)	25	27	12	17	21	20
Age (years), mean ± SD	40.43 ± 11.13	40.42 ± 9.91	33.50 ± 11.78	54.15 ± 7.01	39.86 ± 10.28	34.10 ± 11.6
Female/male number (n) (ratio)	16/9 (1.77)	19/8 (2.375)	8/4 (2)	11/6 (1.83)	15/6 (2.5)	13/7 (1.857)
EDSS mean ± SD	2.75 ± 1.17	2.87 ± 1.29	3.60 ± 0.37	5.68 ± 1.51	1.85 ± 0.95	NA
Disease duration (years), mean ± SD	4.04 ± 3.96	8.5 ± 4.81	4.62 ± 3.19	20.88 ± 13.65	1.85 ± 1.21	NA
Treatment duration (months), mean ± SD	18.00 ± 13.96	30.37 ± 18.69	37.75 ± 22.91	19.00 ± 12.00	NA	NA

*NA* non applicable

### Study Protocol

The study was approved by the local Ethics Committee of the Medical University of Silesia (KNW/0022/KB1/130/12).

After obtaining informed consent, demographic data, Kurtzke’s Expanded Disability Status Scale (EDSS) [24], Athens Insomnia Scale (ASI) and Beck Depression Inventory (BDI) questionnaires were completed in all groups. Head magnetic resonance imaging (MRI) examinations were performed in all MS patients at the beginning of the study, in accordance with standard clinical protocols. The neurological examination was performed by a qualified neurologist using the EDSS before the therapy and after its completion. All MS patients were supplemented orally with melatonin, 5 mg per day, over a period of 90 days. Before and after the melatonin supplementation, the TOS and TAC values in the serum were measured.

### Enzymatic Assays

Ten milliliter samples of venous blood, before and after the 90 days of melatonin supplementation, were collected from the MS patients between 6.00 and 7.00 a.m., centrifuged and frozen until laboratory measurements were performed.

The TOS of the serum was measured using an automated calorimetric method according to Erel [25]. The results were expressed in terms of micromolar hydrogen peroxide equivalent per liter (µmol H_2_O_2_ equivalent/L). The TAC was measured according to Erel [26]. The achieved results were expressed as mmol Trolox equivalent/L.

All laboratory analyses were performed at the Department of Biochemistry in Zabrze, Medical University of Silesia.

### Questionnaires

The sleep conditions were evaluated using the Polish validated reliable version of AIS questionnaire as a self-assesement tool designed to measure sleep difficulty. AIS defines insomnia based on the ICD-10 criteria. AIS consists of eight items inquiring about sleep conditions over the previous month. The first five items assess sleep difficulties with regard to the quantity and quality of sleep: difficulties falling asleep, maintaining sleep, early morning awaking, total sleep duration and sleep quality. The last three items assess the following-day consequences of sleep or daytime symptoms, which may result from insomnia and/or a sleep disorder: subjective well-being, functioning capacity and sleepiness during the day. Each item of the AIS is rated on a scale from 0 (no problem at all) to 3 (a very serious problem) with the maximum score of 24; a score of 6 or more points indicating insomnia. The original validation study demonstrated good internal, test–retest reliability and external validity [27]. Validation of the Polish version AIS was performed in 2011. The internal consistency (Cronbach’s α = 0.90) and the test–retest reliability (r^2^ = 0.92) of the AIS were found to be very satisfactory [28].

The BDI is an instrument for the evaluation of depression [29]. It is composed of 21 items rated on a scale from 0 to 3. Patients with scores between 0 and 9 are not recognized as depressed, scores between 10 and 18 indicate mild to moderate depression, scores above 19 mean moderate to severe depression. The valid Polish version of BDI was applied in our study [30].

### MRI Examination

The MRI was performed in all MS patients at the beginning of the study. The imaging was performed with the use of a General Electric HDx 1,5T scanner (USA). The patients were scanned with a standard head protocol [multiple planes, slice thickness 5 mm, contrast media: Gadovist (Gd)] and additional postcontrast 3DT1 sequences (1 mm slice thickness). An experienced radiologist reviewed the scans and assessed the approximate number of supratentorial and infratentorial plaques in T2 images. The number of enhancing T1 plaques was also reported.

### Statistics

All results expressed as mean ± SEM. Normal data distribution was tested with the Kolmogorov–Smirnov’s test. Comparisons between groups were performed using the Mann–Whitney U test and Wilcoxon test. Differences between means were considered statistically significant at *p* < 0.05. The Pearson correlation analysis was used to estimate the correlation. Results were statistically analyzed using STATISTICA v. 8.0 (StatSoft, Poland).

## Results

The demographic characteristics of the study groups are shown in Table 1.

**Fig. 1:**
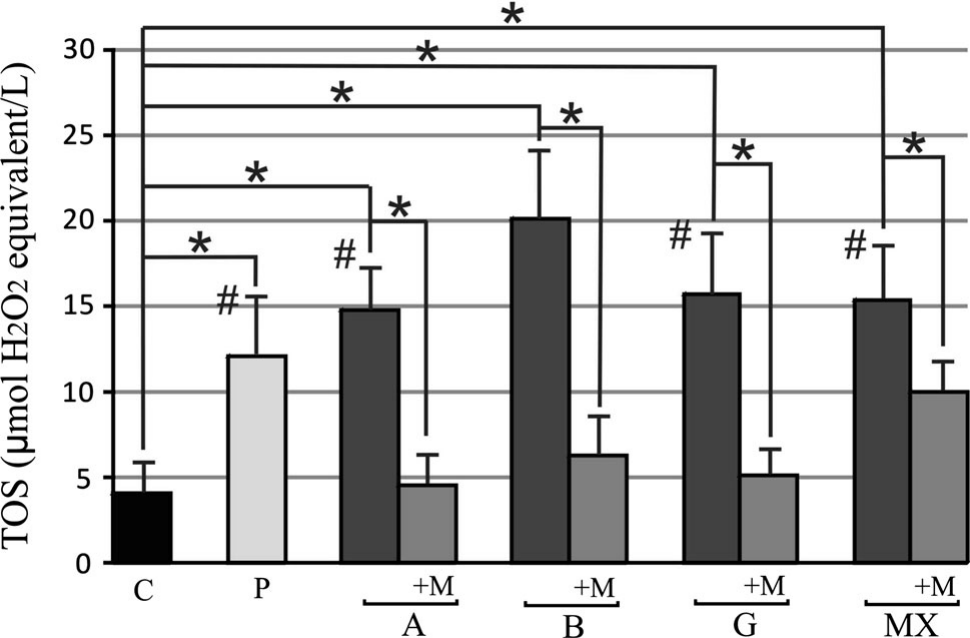
Serum level of total oxidant status (TOS) before and after 3 months 5 mg daily melatonin (M) supplementation in studied groups: *A* (beta-1a interferons treated RRMS group), *B* (beta-1b interferons treated RRMS group), *G* (glatiramer acetate treated RRMS group), *MX* (mitoxantrone treated SP or PR MS group), *P* (immunomodifying pre-treated RRMS group), *C* (control healthy group). Data presented as mean ± SD; *p < 0.05 versus control; ^#^ *p* < 0.05 versus B group

We observed a significant, two to five-fold, increase compared to controls 4.09 ± 1.83 µmol H_2_O_2_ equivalent/L serum level of TOS in all MS patients groups in order P, A, B, G, MX: 12.13 ± 3.81, 14.70 ± 2.44, 20.07 ± 4.51, 15.63 ± 3.86, 15.38 ± 3.71 (µmol H_2_O_2_ equivalent/L). There were statistically significant differences between the studied groups only compared B with other MS groups. After 90-days of the melatonin treatment, the TOS level was significantly lower in all MS-treated groups: 4.55 ± 1.85, 6.26 ± 1.95, 5.15 ± 1.9, 10.00 ± 1.97 (µmol H_2_O_2_ equivalent/L) in groups A, B, G, MX, respectively, but not in group P. The reduction in the TOS level was mostly observed in groups A, B, G (about a 3-fold reduction) (*p* < 0.05) reaching values similar to those in the control group (Fig. 1).

**Fig. 2:**
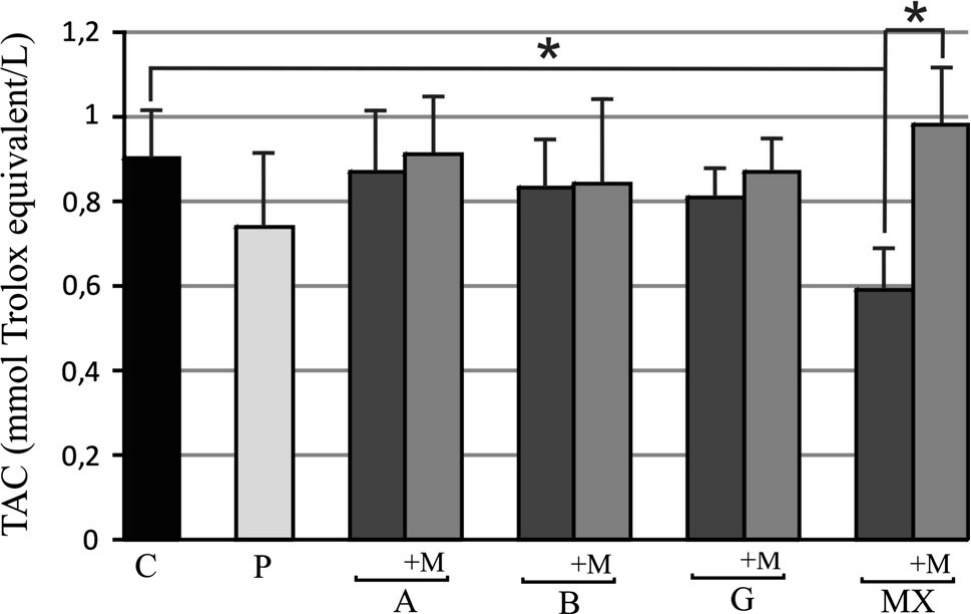
Serum level of total antioxidant capacity (TAC) before and after 3 months 5 mg daily melatonin (M) supplementation in studied groups: *A* (beta-1a interferons treated RRMS group), *B* (beta-1b interferons treated RRMS group), *G* (glatiramer acetate treated RRMS group), *MX* (mitoxantrone treated SP or PR MS group), *P* (immunomodifying pre-treated RRMS group), *C* (control healthy group). Data presented as mean ± SD; *p < 0.05

TAC level was significantly lower only in the MX group compared to the controls: 0.63 ± 0.22 versus 0.90 ± 0.11 mmol Trolox equivalent/L, respectively. After 3 months of the melatonin treatment, an increase in TAC level was observed only in the MX group at 0.98 ± 0.3 mmol Trolox equivalent/L, achieving a similar value to that of the controls (*p* < 0.05). There were no recorded differences between other studied groups, and no significant results were observed after the melatonin treatment in other groups (Fig. 2).

There are negative correlations between the TAC and TOS levels before the melatonin treatment for the MX group: r = −0.67, *p* < 0.05. Those negative correlations were also observed after the 3-month long melatonin supplementation in groups A and B: r = −0.67, r = −0.91, respectively (*p* < 0.05). The TOS and TAC results in the serum after the melatonin treatment for group P were inconsistent, and as such, difficult to interpret, requiring additional studies (data not presented).

Numbers of MRI supratentorial and infratentorial T2 and T1 Gd(+) lesions for studied groups are shown in Table 2.

**Table 2:** Number of MRI supratententorial, infratentorial T2 lesions and T1 Gd(+) lesions for studied groups: A (beta-1a interferons treated RRMS group), B (beta-1b interferons treated RRMS group), G (glatiramer acetate treated RRMS group), MX (mitoxantrone treated SP or PR MS group), P (immunomodifying pre-treated RRMS group)

Group	A	B	G	MX	P
Patients number (n)	25	27	12	17	21
Number of supratentorial T2 lesions (mean ± SD)	23.70 ± 19.36	24.05 ± 14.23	29.00 ± 10.53	31.11 ± 12.44	21.71 ± 13.54
Number of infratentorial T2 lesions (mean ± SD)	2.12 ± 0.3	2.01 ± 1.33	5.33 ± 4.72	3.55 ± 2.53	1.42 ± 0.77
Number of supratentorial T1Gd(+) lesions (mean ± SD)	0.625 ± 0.25	0.21 ± 0.71	0	0	0

A positive relationship between supratentorial and infratentorial number of T2 lesions was reported in both the A (r = 0.7, *p* < 0.05) and B group (r = 0.65, *p* < 0.05). A strong positive correlation between the number of T1Gd(+) lesions and TAC level in group A was observed (r = 0.83, *p* < 0.05). On the other hand, a significant negative relationship between the number of supratentorial plaques and mean TOS level was noticed in groups B (r = −0.66, *p* < 0.05), and MX (r = −0.9, *p* < 0.05). Furthermore, a positive correlation between the mean number of T2 supratentorial lesions and disease duration (r = 0.76, *p* < 0.05) was found in group B.

**Fig. 3:**
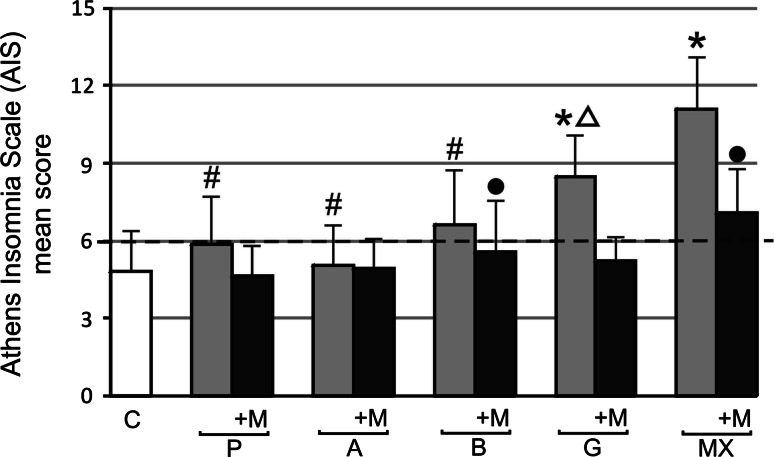
Athens Insomnia Scale (AIS) mean scores before and after 3 months 5 mg daily melatonin (M) supplementation in studied groups: *A* (beta-1a interferons treated RRMS group), *B* (beta-1b interferons treated RRMS group), *G* (glatiramer acetate treated RRMS group), *MX* (mitoxantrone treated SP or PR MS group), *P* (immunomodifying pre-treated RRMS group), *C* (control healthy group). Points above the *dotted line* correspond to insomnia. Data presented as mean ± SD. *p < 0.05 versus control (C); ^•^ *p* < 0.05 after melatonin (M) supplemented group versus before melatonin supplemented group; ^#^ *p* < 0.05 AIS A, B, P group mean score versus MX AIS mean score before melatonin (M) supplementation; ^Δ^ *p* < 0.05 AIS A mean score versus AIS G mean score

AIS mean group score above 6, indicating insomnia, was observed in groups B, G and MX: 6.62 ± 2.88, 8.45 ± 2.07, 11.1 ± 3.25, respectively. AIS mean score was significantly higher in groups G and MX than in controls: 4.83 ± 2.13. After the 3 months of the melatonin treatment, AIS mean scores decreased in groups G and MX, achieving the values of 5.25 ± 1.14 and 7.08 ± 2.39, respectively (*p* < 0.05). MX group AIS mean scores were statistically higher than A, B, and P group mean scores, and the G group mean AIS score was significantly higher than in group A (Fig. 3). An AIS positive correlation in MX groups after and before melatonin supplementation was observed r = 0.7, *p* < 0.05.

**Fig. 4:**
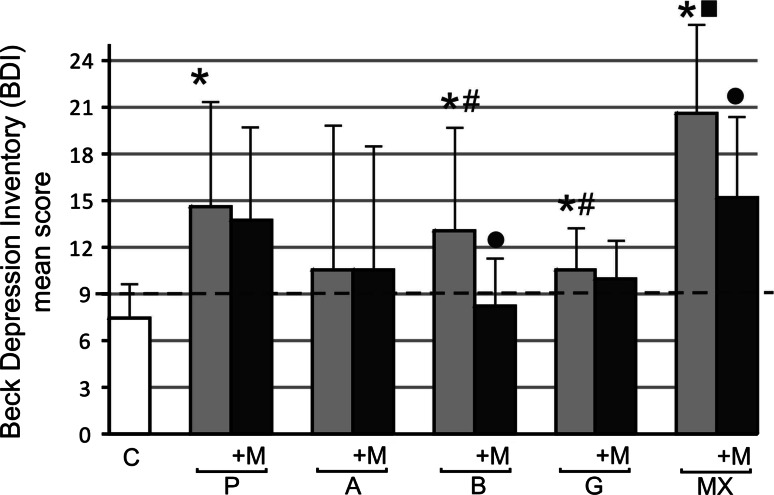
Beck Depression Inventory (BDI) mean scores before and after 3 months 5 mg daily melatonin (M) supplementation in studied groups: *A* (beta-1a interferons treated RRMS group), *B* (beta-1b interferons treated RRMS group), *G* (glatiramer acetate treated RRMS group), *MX* (mitoxantrone treated SP or PR MS group), *P* (immunomodifying pre-treated RRMS group), *C* (control healthy group). Points above the *dotted line* correspond to depression. Data presented as mean ± SD. *p < 0.05 versus control (C); ^•^ *p* < 0.05 after melatonin (M) supplemented group versus before melatonin supplemented group; ^**#**^ *p* < 0.05 BDI B, *G* group mean score versus MX BDI mean score before melatonin (M) supplementation; ^■^ *p* < 0.05 BDI MX mean score versus BDI P mean score

In all MS groups mean scores increased above 9 indicating depression were observed: 14.62 ± 7.06 in group P, 10.54 ± 9.44 in group A, 13.06 ± 6.29 in group B, 10.55 ± 2.88 in group G, 20.58 ± 5.8 in group MX compared to 7.47 ± 2.29 in the control group. Differences in mean BDI scores between groups before melatonin treatment were observed only between P and MX groups (*p* < 0.05). A significantly higher mean BDI score was observed in group MX compared to groups P, B and G (*p* < 0.05) (Fig. 4).

Strong positive correlations were observed only between AIS and BDI mean scores in group A after melatonin treatment compared to group A (r = 0.87, *p* < 0.05) and in group B after melatonin treatment compared to group B (r = 0.72, *p* < 0.05).

## Discussion

The results from our work strongly suggest that MS patients have a higher level of TOS than healthy controls do—both immunomodulatory drug-treated patients and those without such kind of therapy. Many studies have suggested that the pathogenic traits of MS implicate oxidative stress as one of important factors in initiating and perpetuating mechanisms responsible for progressive neurological impairment [4, 7, 31]. Blood antioxidant enzymes: SOD, CAT, and glutathione reductase (GR) activity was found to be elevated in RRMS against SPMS and controls and negative correlation was established between SOD and CAT and EDSS in RRMS [32].

Interestingly enough, in our study there were no significant differences observed between immunomodulatory treated and untreated patients, but the interferon beta-1b patient group had a statistically higher level of TOS than the other immunomodulatory treated groups. Melatonin supplementation over 3 months caused a marked decrease in TOS levels in all MS patient groups achieving immunomodulatory drugs even to controls values in interferons beta and glatiramer acetate groups. Surprisingly, this decrease wasn’t reported in MS patient groups lacking any immunomodifying therapy.

Melatonin upregulates antioxidative defensive systems, including the activities of SOD and GSH-Px, as well as the levels of GSH and these actions further reduce the oxidation state of cells. Experimental evidence supports its actions as an indirect antioxidant when stimulating antioxidant enzymes, its ability to enhance the activities of other antioxidants, and its protection of antioxidant enzymes from oxidative damage [3, 10, 11].

In our research, the TAC was reduced as compared to controls only in the MX-treated group; in all others group TAC was comparable with healthy controls. This result confirms that in the MX group the impairment of antioxidant systems is present. Similarly, lower levels of GSH, a potent endogenous antioxidant for SPMS patients rather than for controls, measured in the fronto-parietal regions of the brain using a magnetic resonance spectroscopy technique were found. The results indicate the presence of oxidative stress in SPMS patients [33]. Conversely, in another study, no difference in the TAC level in serum between SPMS patients and controls was observed [34]. The abovementioned differences are probably associated with different characteristics of the patients studied.

We observed that after the melatonin supplementation TAC level increased significantly in the MX group, but not in other groups. Similarly, Miller et al. [35] reported that TAC level was lower in the MS patient group with long disease duration as compared to controls, and after a 2 week period of 10 mg melatonin supplementation the TAC level increased significantly. Furthermore, 1 month of 10 mg melatonin supplementation caused a statistically significant increase in SOD and GSH-Px activities in erythrocytes in SPMS patients [36].

An interesting positive relationship was noticed in our survey between the number of T1 Gd(+) lesions and TAC level, but statistically significant only in the RRMS interferon beta-1a group. MRI provides an exact index of MS-related inflammatory processes, marking the evolution of the disease through the size and number of Gd(+) lesions and the proportion of T_1_/T_2_ lesions. However, disease progression causes a reduction in inflammatory markers and an increase in oxidative stress parameters leading to neurodegeneration. The number of accompanying enhanced lesions declines, T_2_-weighted lesions stabilize the brain atrophy stemming from cell loss [37]. On the other hand, in our study, significant negative correlations between the number of supratentorial plaques and mean TOS level (in groups B and MX) were found. Furthermore, a positive correlation between the mean number of T2 supratentorial lesions and disease duration was found in group B.

Many abovementioned biochemical parameters in interferons- and glatiramer acetate-treated MS patients were different than in the P and MX patient groups. These observations are very difficult to interpret, because even many years of therapy with these drugs in MS brought no response about detailed mechanisms of their therapeutic actions [38].

It is known that some of effects of melatonin are mediated via the maintenance of mitochondrial oxidative phosphorylation and optimized mitochondria functioning. Disturbances in mitochondrial processes mechanisms are relevant in human MS and animal MS models. Dysfunctional mitochondria are important contributors to damage and loss of both axons and neurons. Observations in animal and histopathological studies suggest that infiltrating leukocytes and activated microglia play a central role in neuronal mitochondrial dysfunction [39]. It was proved that the number of degenerating axons and increased intra-axonal mitochondria also correlated strongly with global measures of disease course, such as total lesion load, spinal cord atrophy, and neurological function in a murine model of MS [40].

It is known that sleep disturbances are more often present in MS patients than in the general population [14–17]. The possible common pathomechanisms shared by MS and sleep disturbances may be connected with circadian rhythm disorders with compromised melatonin secretion reducing input to the suprachiasmatic nucleus due to impaired visual pathways, as well as increased levels of proinflammatory cytokines [41]. Najafi et al. [42] reported that sleep phase syndrome and irregular sleep wake patterns in MS patients with mild and severe fatigue were compared with healthy subjects. Circadian rhythm sleep disorders were significantly higher in MS patients in relation to healthy subjects. In our work, we observed that the majority of MS immunomodulatory-treated patients met the insomnia criteria—mean AIS scores above 6, indicating insomnia, were observed in groups B, G, and MX, while more advanced cases of insomnia were found in MX and G patient groups. This fact might be connected with higher EDSS scores in both groups and a markedly longer disease duration in the MX groups, but no correlations were observed. In other Polish MS population almost 50 % of MS patients complained about sleep disturbances, and sleep disturbances did not depend on either the disability, duration of disease, or its course [43]. In our research, after 3 months of the melatonin treatment, mean AIS scores decreased by about 37 and 35 % in groups G and MX, respectively. An AIS positive correlation in the MX groups before and after the melatonin supplementation was observed. These results strongly suggest that the more advanced insomnia in MX- and G-treated patients could be treated with melatonin with satisfactory results. Melatonin is one of the major regulators of the sleep-wake cycle. There was an association observed between shift work at a young age and the occurrence of MS. Consequences of shift work, such as circadian disruption and sleep restriction, were associated with disturbed melatonin secretion and enhanced proinflammatory responses [19]. Melamud et al. [41] reported significantly lower sleep efficiency in the MS patients connected with dysregulation of melatonin secretion in MS patients, which may be influenced by treatment with interferons beta. Exogenous melatonin has somniferous properties in normal subjects and can improve sleep quality in several clinical conditions. Recent studies have shown that melatonin may play a role in improving sleep in patients [12].

In our study we evaluated depression in MS patients and the results demonstrate that all our MS patients suffer from depression with the moderate level in MX-treated patient group. Prevalence of depression could vary slightly with BDI-cut off scores or with the tools for assessment of depression in MS-affected people. In Chwastiak et al. [44] study clinically depressive symptoms were observed in 41.8 % of the subjects. Significantly higher levels of depression in MS patients compare to the control group was observed in previous Polish study. The mean BDI score in Papuc et al. [45] paper was 14.81 ± 8.80 versus 5.20 ± 4.89; *p* < 0.05. Moroeover, many recent studies showed that increased incidence of depression in MS patients is associated with IFNβ therapy [46, 47]. Contrary, Kirzinger et al. [48] indicated no differences in mean BDI score in MS patients after over 48 months of disease-modifying therapy. In our study we did not observe any differences in mean BDI scores between pre-treated patients and patients treated with interferons or glatiramer acetate, but there were differences observed between pre-treated and mitoxantrone treated patients. Some of the mixed results as to the effects of different treatments in this study may be confounded by depression levels. Furthermore, depression is associated with decreased levels of serotonin, which is a precursor for NAS and melatonin. Given that melatonin regulates immune cell responses relevant to MS [49], depression levels could be mentioned as possible confounding factor in sleep quality evaluated in our study.

Our results suggest that melatonin could be used with a satisfactory outcome in MS patients with more advanced insomnia. Its ability to regulate the circadian rhythm in a disease associated with sleep disturbances, such as MS, could be beneficial. Therefore, more investigation is needed to ascertain the exact role of melatonin in the treatment and pathophysiology of MS, taking into account the molecular basis and including the crucial mitochondrial mechanisms.
